# 1210. Recommendations for Screening and Diagnosis of Chagas Disease in the United States

**DOI:** 10.1093/ofid/ofab466.1402

**Published:** 2021-12-04

**Authors:** Colin Forsyth, Jen Manne-Goehler, Jen Manne-Goehler, Caryn Bern, Jeffrey Whitman, Morven S Edwards, Natasha Hochberg, Rachel Marcus, Norman Beatty, Yagahira Castro, Christina Coyle, Paula Stigler Granados, David H Hamer, James Maguire, Robert Gilman, Sheba Meymandi

**Affiliations:** 1 Drugs for Neglected Diseases initiative, New York, New York; 2 Brigham and Women’s Hospital, Boston, MA; 3 University of California, San Francisco, San Francisco, California; 4 Baylor College of Medicine, Houston, Texas; 5 Boston Medical Center, Boston, MA; 6 Medstar Union Memorial Hospital, Washington, District of Columbia; 7 University of Florida, Gainesville, Florida; 8 Johns Hopkins University, Baltimore, Maryland; 9 Albert Einstein College of Medicine, New York, New York; 10 Texas State University, San Marcos, Texas; 11 Boston University, Boston, MA; 12 Brigham and Women’s Hospital and Harvard Medical School, Boston, Massachusetts; 13 Olive View-UCLA Medical Center, Sylmar, California

## Abstract

**Background:**

Over 300,000 people in the United States are infected with *Trypanosoma cruzi,* the protozoan parasite that causes Chagas disease (CD). Only about 1% of estimated U.S. cases have been identified, usually through blood donor screening, and most people are unaware they have the infection. Screening is critical for increasing case detection and ensuring patients receive appropriate and timely care, but awareness of CD management strategies among healthcare providers is low. Diagnostic guidelines for CD in the United States are needed to increase provider-directed screening and diagnosis.

**Methods:**

Screening recommendations were prepared by the U.S. Chagas Diagnostic Working Group, which consists of clinicians, researchers, and public health experts involved in CD programs. The group agreed on six main questions based on the PICO method (Population, Intervention, Comparison, and Outcome). Subgroups discussed each and proposed initial recommendations, which were then shared and validated within the larger group. The recommendations used the GRADE methodology, assigning two sets of ratings: 1) strength of the recommendation, and 2) quality of the evidence.

**Results:**

The group recommended screening anyone who was born or lived for >6 months in South America, Central America and Mexico (Figure 1). Recent community-based studies found a prevalence of 1-3.8% in this population. Within this population, having a family member with CD, or having clinical conditions suggestive of CD, including electrocardiographic abnormalities, suggest an elevated risk. Screening women of childbearing age and infants born to seropositive women is important for preventing congenital transmission. Test performance may vary depending on several factors, including whether patients are from South America, Central America or Mexico. Confirmation therefore requires positive results on at least two serological tests based on different antigens or formats, in line with Pan American Health Organization (PAHO) recommendations. Once CD is confirmed, patients should receive an electrocardiogram and echocardiogram to monitor for development of cardiac complications.

**Conclusion:**

These CD screening recommendations are meant to be a resource for U.S. healthcare providers to simplify testing of at-risk patients.

**Disclosures:**

**Jen Manne-Goehler, MD, DSc**, Regeneron (Individual(s) Involved: Self): Scientific Research Study Investigator **Caryn Bern, MD, MPH**, **UpToDate (Wolters Kluwer**) (Other Financial or Material Support, Author Royalties)

